# Successful Aging Across Middle Versus High-Income Countries: An Analysis of the Role of eHealth Literacy Associated With Loneliness and Well-Being

**DOI:** 10.1093/geront/gnae170

**Published:** 2024-12-14

**Authors:** Loredana Ivan, Hannah R Marston, Vishnunarayan Girishan Prabhu, Franziska Großschädl, Paula Alexandra Silva, Sandra C Buttigieg, Halime Öztürk Çalıkoğlu, Burcu Bilir Koca, Hasan Arslan, Rubal Kanozia, Matthew H E M Browning, Shannon Freeman, Sarah Earle

**Affiliations:** The National University of Political Studies and Public Administration (SNSPA), Bucharest, Romania; School of Health, Wellbeing, and Social Care, The Open University, Milton Keynes, UK; Department of Industrial and Systems Engineering, University of North Carolina, Charlotte, North Carolina, USA; Institute of Nursing Science and Age and Care Research Group, Medical University Graz, Graz, Austria; Department of Informatics Engineering (DEI), Centre for Informatics and Systems (CISUC), Faculty of Science and Technology, University of Coimbra, Coimbra, Portugal; Faculty of Health Sciences, Department of Health Systems Management and Leadership, University of Malta, Tal-Qroqq, Msida, Malta; Department of Educational Sciences, Canakkale Onsekiz Mart University, Çanakkale, Turkey; Department of Educational Sciences, Canakkale Onsekiz Mart University, Çanakkale, Turkey; Department of Educational Sciences, Canakkale Onsekiz Mart University, Çanakkale, Turkey; Department of Mass Communication and Media Studies, Central University of Punjab, Bathinda, India; Department of Parks, Recreation, and Tourism Management, Clemson University, Clemson, South Carolina, USA; School of Nursing, University of Northern British Columbia, Prince George, British Columbia, Canada; School of Social Sciences, Nottingham Trent University, Nottingham, UK

**Keywords:** COVID-19, Cross-cultural studies, e-Health, Successful aging, Technology

## Abstract

**Background and Objectives:**

“Successful aging” concerns the process of growing older while maintaining physical, cognitive, and social well-being, emphasizing independence for overall satisfaction and quality of life. We investigate the impact of eHealth literacy on reducing loneliness and sustaining well-being during the pandemic, comparing middle- and high-income countries.

**Research Design and Methods:**

Online surveys were conducted between April 4, 2020, and September 30, 2021, collecting responses (*N* = 2,091) from medium- and high-income countries in Europe, Asia, and North America. *T*-tests and ANOVAs were used to test how sociodemographic predictors were associated with differences in e-Health literacy, loneliness, and well-being.

**Results:**

Respondents from high-income countries reported significantly higher well-being scores than those from middle-income countries and respondents from high-income countries had significantly higher e-HEALS (e-Health literacy) scores compared to middle-income countries. No significant difference was observed in loneliness scores between high-income and middle-income country respondents. Well-being is associated with age, with younger adults (18–29 years) and those aged 40+ reporting higher levels. Higher education and income are linked to greater well-being. Gender differences are observed, with females and those with a partner reporting higher well-being. In middle-income countries, higher education levels are more linked to loneliness, while in higher-income countries, loneliness is observed across education levels.

**Discussion and Implications:**

Future interventions by governments and policymakers should consider intersectionality in e-Health planning and offer digital literacy and digital skills training to those with lower education levels.

The current study investigates the relationship between e-Health literacy, perceived loneliness, and well-being for the older (60+) and younger age groups, during the coronavirus disease 2019 (COVID-19) pandemic. We focus on the differences between middle- and high-income countries in the context of a dominant “successful aging” discourse. Financial security is indicated by lay people from various countries as a precondition of successful aging (e.g., Jensen et al., 2018). Although the country’s resources provide the terrain for policies associated with successful aging rhetoric, no study that we are aware of explicitly includes comparisons between countries with different economic development. From this perspective, the current study tries to fill a gap in the already existing literature.

Since the concept of successful aging was introduced (Havighurst, 1961), it has been of great interest among gerontology scholars. Successful aging rhetoric has highly influenced public discourse on later life for the past three decades (Bülow & Söderqvist, 2014; Thuesen et al., 2023), since the launch of Rowe and Kahn’s programmatic papers (1987, 1997). It brings to the forefront the vulnerabilities and risks of old age through preventive strategies, the normativity of discourses pertaining to the aging process (Baltes & Carstensen, 1996), subject to interpretations and criticism (Jones & Higgs, 2010; Katz & Calasanti, 2015). Successful aging describes a process developing over time and it is not related to people’s chronological age, involving the positive as well as negative aspects associated with getting old (Pruchno, 2021).

Successful aging (Rowe & Kahn, 1987) has been operationalized by combining three dimensions: (1) probability of disease and disease-related disability; (2) functional and cognitive capacity; (3) engagement and participation in social life; that is, the potential for interaction and social contact. Over the years, the successful aging model (Bülow & Söderqvist, 2014) has been criticized: the optimization and enhancement of the aging individual opens the doors to biomedicine and the influence of a neoliberal political discourse (Katz, 2013; Katz & Marshall, 2003), placing responsibility on the individual for solving the “problems” of aging (Hsu & Jones, 2012). However, using a multidimensional model of successful aging (Rowe & Kahn, 1987, 1997), which emphasizes the importance of social and environmental factors, and the heterogeneity of the aging population, it may be possible to modify the extrinsic factors associated with age decline and generate positive change (Rowe & Kahn, 1997, p. 437) and well-being in later life. A revised model of successful aging (Rowe & Kahn, 2015) considers social factors and technology use, the latter being pivotal to understanding the challenges of the aging process in the 21st century.

Betlej (2023), investigated the factors influencing older adults’ successful aging, and ascertained the independent use of technologies that played an important role for adults older than 60 years. Technology-based solutions, such as video calls (e.g., Facetime, WhatsApp video calls, Zoom) and online chat groups (e.g., Facebook Messenger) have been used to reduce loneliness and maintain well-being later in life (Hwang et al., 2020; Tilburg et al., 2021). Moreover, the adoption of technology was instilled to mitigate against the challenges and negative outcomes of the pandemic lockdowns (Burholt et al., 2020; Vargo et al., 2021). There were various challenges and negative outcomes encountered by people and included receiving their education and learning (Shah et al., 2020) and limited access to healthcare and its wider ecosystem (Ting et al., 2020). Additional concerns relating to isolation and its impact on mental health resulting in wider concerns during the pandemic (Williams et al., 2020) were heightened together with seeking out virus information and identification of transmission through digital apps. For example, the UK devolved nations rolled out a digital app for residents to keep up to date on whether they had come into contact with a person who had tested positive for COVID-19 (Marston et al., 2022). To alleviate these challenges and negative outcomes technology was deployed as a panacea (Vargo et al., 2021), where the Internet and access to technology were universal (Fisk et al., 2020; Marston & Kowert, 2023). During 2020, technology use and associated platforms increased as Scholmann and colleagues identified through a study conducted amongst 1,374 U.S. residents who reported to use various methods to communicate. Including text messaging (43%), voice calls (36%), social media (35%), and video calls (30%) (2020). Technologies such as these facilitated greater opportunities for people geographically dispersed to maintain social connections with their friends, family and work colleagues, playing a pivotal role during lockdowns (Shah et al., 2020).

Other technologies have been designed to treat and monitor people’s health, thus enhancing their cognitive and social abilities (see Shah et al., 2021 for a meta-analysis). There is an intertwinement between successful aging and social support for older adults. Technology is seen as an important mediator, especially in connecting individuals and reducing loneliness. People need to be technologically skilled to convert health information received online through digital technologies into personal interventions, a process requiring e-heath skills. Moreover, the successful aging rhetoric applies to more upper-middle-class individuals from affluent backgrounds and high-income countries and raises some challenges for vulnerable older people in low- and middle-income countries (Ivan & Cutler, 2021).

## Loneliness

During the COVID-19 pandemic, there was a marked overall increase in the use of technologies to reduce loneliness (Marston et al., 2023). For example, half of the adult population in the United States declared in 2021 (Pew Research Center, 2021) that their personal lives have changed in a major way during the COVID-19 pandemic, and 52% referred to the use of new technologies as a major change in their lives. In high-income countries, such as the United States, older adults turned to digital tools to alleviate isolation, with approximately 75% of older adults using technology to stay connected with family and friends (Pew Research Center, 2022; Sen et al., 2022). This surge in ICT use also helped address daily tasks like grocery shopping and healthcare access (Ericsson, 2021). Similarly, across middle-income countries, the pandemic accelerated mobile phone and Internet usage among adults (GSMA, 2023). Digital financial services such as mobile payments saw a surge in adoption, particularly in African and Southeast Asian nations, where adults increasingly relied on technology for transactions and healthcare via telemedicine (Agur et al., 2020; Mahmoud et al., 2022). Although the digital divide remains a challenge, the pandemic spurred rapid technological shifts across regions, reshaping access to essential services and reducing isolation (Banga & te Velde, 2020).

For vulnerable groups, such as older adults, governmental lockdown directives were felt with greater intensity, leading to an increased reliance on technology for communicating with the outside world (Marston et al., 2023). Limited interactions and engagement with one’s social network are known to increase loneliness in older people and pose a risk to physical (Holt-Lunstad et al., 2017) and mental (Miyawaki, 2015) health, such as cardiovascular disease, reduced quality of life, and depression (Courtin & Knapp, 2017).

In this article, we refer to social loneliness and the perception people have of their own situation. Social loneliness is described as a subjective experience of deficit in social relations in a qualitative or quantitative way (De Jong-Gierveld, 1987). We acknowledge the fact that people with few social contacts might not feel lonely. Loneliness (Burholt et al., 2020) refers to the discrepancy between an individual’s desire for social connection and access to, or experience of, social connectivity (Perlman & Peplau, 1981). Studies show that later life is accompanied by a decrease in the number of friends and new acquaintances. However, adults who are highly educated and affluent maintain more frequent and intense social relations, whereas adults with lower education, or cognitive decline, are more at risk of loneliness (Pruchno et al., 2010).

Studies investigating social resources as a key component of successful aging did not measure loneliness, but rather the frequency of, and the number of people with whom older adults socially engage with ([Bibr CIT0087][Bibr CIT0087]). Social resources are treated as a proxy for social loneliness, as loneliness is generally known to be an important factor in physical and cognitive decline (Perissinotto et al., 2012). The greater risk of severe illness and/or mortality among older adults due to COVID-19, resulted in an increased risk of loneliness in this age group (Brooks et al., 2020). Without the necessary technical skills, equipment and support, loneliness poses a risk, particularly among older adults, which can be exacerbated when social distancing is required.

Few studies (Czaja et al., 2021) have investigated older people’s loneliness in researching successful aging and the role of perceived social support. The COVID-19 pandemic brought into the spotlight the importance of situational factors in the availability of social resources. Limitations and recommendations to meet and interact with people while socially distancing were instilled in many countries (with variation in severity of restrictions across countries). It became relevant to research how people experienced situations—in terms of perceived loneliness, rather than to investigate the frequency of social interactions and the number of contacts.

People’s ability to use technologies to alleviate loneliness and to find relevant health information is not equally distributed. Research suggests that vulnerabilities during the COVID-19 pandemic reproduced classic social inequalities: the oldest-old, with poor digital skills, from less economically developed countries, became more isolated and faced an increase in various types of risks (Ivan & Cutler, 2021).

## E-Health Literacy

It is generally recognized that e-Health resources play a growing role in the way individuals from different age groups, particularly the older population, manage their health and connect with others, for example in sharing health experiences and accessing health services as well as making informed health decisions (Cheng et al., 2020). Older adults are a vulnerable group, often lacking the appropriate digital literacy (Marston et al., 2024) to access and use online health information and to make productive use of it. Digital literacy is defined as a combination of skills and capabilities that help people be effective online in searching for information, evaluating and sharing information, and using the appropriate digital tools for different needs and goals (JISC, 2014).

During the COVID-19 pandemic, there was a rise in online health information-seeking behavior among older adults to understand and manage their health independently (Brørs et al., 2020). E-Health literacy is described as the ability to seek out, and appraise health information from electronic sources, to apply acquired knowledge, and to address or solve a health problem (Norman & Skinner, 2006). Health literacy affects the way individuals maintain their cognitive and physical functionalities (Liu et al., 2020). In the case of older adults, e-Health literacy has been operationalized as “actively searching for necessary health information using electronic media, exchanging real-time information, and promoting one’s health by utilizing and sharing it” (Jung et al., 2022, p. 11).

Developing e-Health literacy skills and knowledge to promote healthy aging is a priority of the World Health Organization (WHO) (2022). Because of the growing amount of health information accessible via the Internet and a growing older population, it is important to investigate the concept of e-Health literacy in older adults. This notion is especially important because older adults are part of the “digital divide” phenomenon (Fox & Connolly, 2018; Marston et al., 2023), a digital gap reproducing social inequalities between different groups of people (e.g., the older and the young ones) produced by a lack of coordination between rapid technological development and people’s appropriation of the new digital technologies (Freeman et al., 2022). A recent systematic review (Oh et al., 2021) discusses the current evidence relating to e-Health literacy since 2009 (Oh et al., 2021), specifically focusing on older adults across several countries including the United States, Germany, China, Italy, Sweden, Canada, Iran, and Bangladesh. Overall, 16 studies in this systematic review were identified in using the eHEALS scale by Norman and Skinner (2006). Moreover, this review highlights the paucity in which eHEALS has previously been used in understanding e-Health literacy and its role in older people’s well-being across geographically dispersed populations and countries during the COVID-19 pandemic (Lee et al., 2020). Özkan et al. (2022) suggested that e-Health literacy could have reduced people’s feelings of loneliness and distress. Ghazi et al. (2023) indicated that by increasing e-Health literacy during the COVID-19 pandemic, older people reduced their psychological distress and maintained a good health status.

This paper presents data on the role of e-Health literacy in reducing loneliness and maintaining older adults’ well-being during the pandemic, with a comparison between high- and middle-income countries. While financial constraints are important subjective well-being predictors (Hsu, 2010), they have been treated as individual factors. However, financial restrictions could be approached as a structural factor, and we lack studies investigating older adults from countries with different levels of economic development. The current study aims to fill this gap by comparing middle- and high-income countries on subjective well-being. This work aims to answer the following research questions (RQ):

- RQ1. What were the relations between e-Health literacy, perceived loneliness, and well-being for the older population during the COVID-19 pandemic?
- RQ2. What were the main sociodemographic predictors influencing eHealth literacy, perceived loneliness, and well-being in different countries?

The data here are novel because of the diverse multisite and international data sets collected during the COVID-19 pandemic. This data contributes to several academic fields of gerontechnology, literacy, well-being, gerontology, and global policy strategies, influencing the regional discourse such as the WHO (2022).

## Method

This work is part of a larger project and data set (Marston et al., 2020), presenting findings based on a subset of data from across the sites, in the context of middle- and high-income countries. We explore the role of sociodemographic factors (age group, gender, education level, income, marital and employment status, type of community) alongside country income levels (middle vs high-income) on outcomes (e-Health literacy, well-being, and loneliness). Employing a life course perspective (Elder, 1985), data collection followed a socioecological model (Bronfenbrenner, 2005), allowing age categories to be created after the data collection process.

## Ethical Approval

The study was initially approved by the Human and Research Ethics Committee (HREC) at the Open University [HREC/3551/Marston]. The UK/English version of the survey was deployed on April 4, 2020, and this resulted in consortium partners contacting the PI requesting to deploy their survey versions. A study protocol (Marston et al., 2020) detailing the breakdown of ethical site approvals, survey development, backward translation, and deployment dates (Wave 1). In the autumn of 2020, two more countries (the United States and Canada) joined the Consortium with respective surveys deployed in 2021 (Wave 2).

## Design and Procedure

Recruitment (Marston et al., 2020) for all surveys employed a social media (Twitter and Facebook) approach with a generic website link to the project website (Health and Wellbeing SRA, 2020), enabling respondents to choose the appropriate country/language survey. Given the challenges of recruiting research participants during a pandemic, consortium members employed additional measures, including sharing the generic link across individual mailing lists and personal social media networks, and a purposive snowball sampling approach was also employed (Benfield & Szlemko, 2006). The snowball approach, utilized researchers’ social networks, reached individuals who might have been difficult to reach through alternative sampling methods, particularly considering the limitations imposed by the pandemic. Snowballing sampling methods, including virtual snowballing, are commonly used in challenging situations (Browne, 2007), and facilitated by the researchers’ networks, this was considered appropriate for recruiting a diverse range of participants from multiple countries, and age categories.

For each new site/language, the survey underwent a backward translation process and, where necessary, amendments to language were made for cultural context (Marston et al., 2020) between the site lead and the PI. Ethical approval was sought from the site’s institutional ethics committee or board, and this documentation was shared with the PI to update the primary governing committee. Generated through the Qualtrics software program, an anonymous link was used for each survey for deployment purposes.

## Measurements and Statistical Analysis

The surveys included eight sections, as described in the protocol (Marston et al., 2020) and presented in Supplementary Material. After data collection, respondents’ data with missing values for the UCLA Loneliness, Psychological Well Being (PWB) Scale, eHEALS (The eHealth Literacy Scale), or sociodemographic questions were identified and removed before data analysis (Supplementary Figure S1). Three dependent variables (Loneliness, well-being, and eHealth Literacy), together with three validated measures (UCLA Loneliness, PWB scale, and the eHEALS), formed the measures. The UCLA Loneliness Scale v3 (Russell, 1996) comprises a 20-item scale (1–5 Likert), and there are five questions that need to be reverse coded (scores range from 20 to 80), with higher scores indicating greater loneliness. The eHEALS (Norman & Skinner, 2006) is a self-report questionnaire developed to measure individuals’ perceived skills and comfort in using digital health information to make informed health decisions. This 8-item scale is rated on a 5-point Likert scale (scores range from 8 to 40), with higher scores indicating greater eHealth literacy. eHEALS assess factors including the ability to find, understand, and evaluate online health information.

The PWB 18-item scale (Ryff & Keyes, 1995) is a short version of the original PWB scale, designed to efficiently assess various dimensions of psychological well-being. The scale includes a subset of items from the full scale, covering key aspects such as autonomy, environmental mastery, personal growth, positive relations with others, purpose in life, and self-acceptance. Participants rate their agreement with each item on the Likert scale, and the scores are calculated to obtain a total score for psychological well-being, with higher scores indicating higher levels of well-being. Although some sociodemographic questionnaire responses were adapted based on the countries to align with the local language, the validated measures were only strictly translated. We did not examine measurement invariance since the questionnaire was only translated.

Overall, 3,244 participants started the survey of which 1,928 (60%) participants completed the survey leaving 1,316 (40%) participants who partially completed the survey which were not considered for the analysis. The bivariate analysis evaluated how various sociodemographic predictors (income status, age, gender, education, marital status, employment, and type of community) affected outcomes measured on continuous scales (UCLA 20 Loneliness Score, Psychological Well-being Score, and e-Heals Score) across and within middle/high-income countries. A two-sided one-way ANOVA and independent sample *t* tests were conducted based on the number of levels for the predictor. Tukey post hoc tests identified differences between groups in the case of significant omnibus ANOVA tests. A Pearson’s correlation test was performed to evaluate the relationship between e-Health literacy, loneliness, and well-being across high-income (HI) and middle-income (MI) countries. An alpha level of *p* < .05 indicates statistical significance.

## Findings

Table 1 provides an overview of the results of Wave 1 (collected in 2020) and Wave 2 results (collected in 2021) with a breakdown of survey response rates and income levels. Respondent characteristics (Table 2) show the fewest respondents (60+ years) and the most (18–29 years) age groups. Overall, 70% of respondents were female, and only <2% reported being nonbinary or preferred not to answer across both groups. Approximately ~90% of respondents had a university degree or higher level of education (Bachelor’s, Master’s, or PhD), and >50% of participants were married/had a partner. >60% of participants were employed, and ~50% reported residing in a metropolitan/city.

**Table 1.** Survey Response Rate

Income	Site	Language	Date survey opened (DD.MM.YYYY)	Date survey closed (DD.MM.YYYY)	Sample (*n* = 3,244)	Percent of sample %
High income	Austria	German	05.06.2020	05.09.2020	240	7.4
High income	Canada	English	06.01.2021	09.31.2021	209	6.4
High income	France	French	12.05.2020	12.08.2020	135	4.2
High income	Germany	German	04.06.2020	04.09.2020	329	10.1
Middle income	India	English	31.05.2020	31.08.2020	320	9.9
Middle income	India	Hindi	31.05.2020	31.08.2020	49	1.5
High income	Malta	English	19.05.2020	19.08.2020	103	3.2
High income	Portugal	Portuguese	29.05.2020	29.08.2020	37	1.1
Middle income	Romania	Romanian	20.04.2020	20.07.2020	447	13.8
High income	Singapore	English	17.05.2020	17.08.2020	82	2.5
High income	Singapore	Mandarin	13.05.2020	13.08.2020	17	0.5
High income	Spain/South America	Catalan/Spanish	04.05.2020	04.08.2020	382	11.8
Middle income	Turkey	Turkish	29.06.2020	29.09.2020	108	3.3
High income	UK	English	03.04.2020	04.07.2020	548	16.9
High income	USA	English	03.29.2021	06.18.2021	238	7.3

**Table 2.** Respondent Characteristics

	HI *n* = 1,450 (%)	MI *n* = 478 (%)
Age group (years)		
18–29	375 (25.9)	221 (46.2)
30–39	342 (23.6)	123 (25.7)
40–49	327 (22.6)	98 (20.5)
50–59	210 (14.5)	22 (4.6)
60 and older	196 (13.5)	14 (2.9)
Gender		
Male	394 (27.2)	130 (27.2)
Female	1,029 (71.0)	342 (71.5)
Nonbinary	11 (0.8)	2 (0.4)
Choose not to answer	16 (1.1)	4 (0.8)
Education level		
Primary or less than high school	35 (2.4)	11 (2.3)
High school	112 (7.7)	37 (7.7)
College diploma/some college or university	279 (19.2)	31 (6.5)
Bachelor’s degree/professional degree	410 (28.3)	117 (24.5)
Masters	387 (26.7)	206 (43.1)
PhD	227 (15.7)	76 (15.9)
Marital status		
Having a partner/married	744 (51.3)	237 (49.6)
Divorced/separated	94 (6.5)	24 (5.0)
Widowed	42 (2.9)	4 (0.4)
Single	514 (35.4)	211 (44.1)
Prefer not to say	56 (3.9)	4 (0.8)
Employment status		
Employed	1,099 (75.8)	284 (59.4)
Retired	117 (8.1)	7 (1.5)
Not employed (out of a job or due to other reasons)	234 (16.1)	187 (39.1)
Type of community (residence)		
Metropolitan/city	667 (46.0)	243 (50.8)
Suburban	294 (20.3)	104 (21.8)
Small town	289 (19.9)	54 (11.3)
Rural area	200 (13.8)	77 (16.1)

*Notes*: HI= = high income; MI= = middle income.

## High-Income Versus Middle-Income Countries

Comparing loneliness, well-being, and eHEALS scores between HI and MI countries (Table 3), findings show the respondents from HI countries (94.3 ± 14.4) reported significantly higher (*t*_1926_ = 1.90, *p* value = .05) well-being scores than those respondents from MI countries (92.9 ± 13.5). Respondents from HI countries (32.1 ± 6.2) reported significantly higher (*t*_1926_ = 3.37, *p* value < .01) eHEALS scores than those from MI countries (30.9 ± 5.4).

**Table 3.** Factors Affecting Well-being and Loneliness

	HI	MI	HI	MI	HI	MI	Correlation (*r*) 95% CI	*p* Value
	Well-being		Loneliness		eHealth			
Age group (years)								
18–29	89.5 ± 13.6	87.5 ± 13.8	47.0 ± 5.1	47.9 ± 5.1	31.4 ± 5.9	30.6 ± 5.5	—	—
30–39	91.6 ± 14.5	93.9 ± 11.2^*^	46.8 ± 5.2	46.2 ± 4.8	31.5 ± 6.2	31.8 ± 5.3	—	—
40–49	96.1 ± 14.4^*^	95.9 ± 13.8^*^	46.1 ± 4.7	45.6 ± 4.7	32.2 ± 5.7	30.9 ± 4.9	—	—
50–59	95.9 ± 13.5^*^	97.1 ± 11.5^*^	45.9 ± 5.2	44.9 ± 4.4^*^	33.7 ± 6.4^*^	31.1 ± 6.4	—	—
60 and older	99.8 ± 13.6^*^	96.1 ± 11.8^*^	45.5 ± 5.6	43.7 ± 2.8^*^	32.3 ± 6.8	31.7 ± 6.0^*^	—	—
Gender								
Male	93.2 ± 15.1	90.1 ± 14.1	46.6 ± 5.2	47.7 ± 4.6	31.5 ± 6.0	31.1 ± 5.3	—	—
Female	94.9 ± 13.9^*^	93.6 ± 12.8^*^	46.3 ± 5.3	45.2 ± 4.8^*^	33.2 ± 5.4^*^	30.9 ± 5.4	—	—
Education level								
Primary or less than high school	92.6 ± 14.7	86.9 ± 16.5	46.7 ± 4.5	49.1 ± 3.7^*^	31.0 ± 6.0	31.2 ± 4.9	—	—
High school	93.1 ± 13.3	88.8 ± 12.9	46.0 ± 5.3	47.8 ± 5.9	30.3 ± 6.5	30.6 ± 5.3	—	—
College diploma/some college or university	91.9 ± 15.6	87.5 ± 11.1	47.4 ± 5.6^*^	46.7 ± 5.1	31.1 ± 6.4	31.1 ± 6.0	—	—
Bachelor’s degree/professional degree	93.3 ± 13.5	92.5 ± 13.5	46.5 ± 5.1	47.0 ± 5.0	31.4 ± 6.0	31.0 ± 5.4	—	—
Masters	97.9 ± 13.5^*^	94.7 ± 12.9^*^	45.7 ± 5.3	46.6 ± 5.0	32.7 ± 5.9^*^	31.1 ± 5.5	—	—
PhD	97.1 ± 13.8^*^	94.2 ± 13.9^*^	45.5 ± 5.4	46.1 ± 3.7	34.4 ± 5.1^*^	30.3 ± 5.0	—	—
Marital status								
Having a partner/married	96.5 ± 13.9^*^	96.7 ± 12.1^†^	45.2 ± 5.1^†^	45.5 ± 4.9^†^	32.6 ± 6.2^*^	31.1 ± 5.4	—	—
Divorced/separated	95.7 ± 13.7^*^	93.1 ± 14.7^*^	47.5 ± 5.6	47.1 ± 4.7	32.4 ± 6.6^*^	31.3 ± 4.0	—	—
Widowed	95.4 ± 15.6^*^	—	45.6 ± 5.3	—	31.0 ± 6.1	—	—	—
Single	91.1 ± 14.1	89.0 ± 12.9	47.8 ± 5.2	48.0 ± 4.9	31.4 ± 6.0	30.8 ± 5.5	—	—
Employment status								
Employed	94.6 ± 14.3^*^	95.2 ± 12.9^†^	46.3 ± 5.2	45.3 ± 4.8^†^	32.5 ± 6.0^*^	31.0 ± 5.5	—	—
Retired	98.7 ± 13.3^†^	—	45.0 ± 5.5^*^	—	31.4 ± 6.0	—	—	—
Not employed	91.6 ± 14.2	89.8 ± 13.4	47.6 ± 5.6	48.0 ± 4.9	31.2 ± 7.3	30.9 ± 5.3	—	—
Type of community								
Metropolitan/city	94.1 ± 14.0^*^	94.6 ± 13.8^*^	46.4 ± 5.2	46.2 ± 5.0	31.7 ± 5.9	31.3 ± 5.5	—	—
Suburban	94.5 ± 14.1^*^	93.3 ± 12.6^*^	46.7 ± 5.4	48.0 ± 4.4	32.4 ± 5.7	30.5 ± 5.5	—	—
Small town	91.6 ± 15.5	90.4 ± 10.8	46.5 ± 5.5	47.6 ± 3.7	32.2 ± 6.5	31.2 ± 5.3	—	—
Rural area	97.8 ± 13.3^†^	89.8 ± 14.1	45.8 ± 5.4	46.7 ± 5.6	32.5 ± 7.1	30.9 ± 5.1	—	—
HI vs MI countries								
Average	94.3 ± 14.4	92.9 ± 13.5	46.4 ± 5.4	46.8 ± 5.0	46.4 ± 5.4	46.8 ± 5.0	—	—
*p* Value	.05	—	.16	—	<.01	—	—	—
High income								
Loneliness-Well-being	—	—	—	—	—	—	−0.59 (−0.62 to −0.55)	<.01
Loneliness—eHealth	—	—	—	—	—	—	−0.13 (−0.18 to −0.07)	<.01
Well-being—eHealth	—	—	—	—	—	—	0.21 (0.16–0.26)	<.01
Middle income								
LonelinessWell-being	—	—	—	—	—	—	−0.59 (−0.59 to −0.46)	<.01
Loneliness—eHealth	—	—	—	—	—	—	−0.07 (−0.16–0.02)	.12
Well-being—eHealth	—	—	—	—	—	—	0.15 (0.06–0.24)	<.01

*Notes*: CI = confidence interval; HI = high income; MI = middle income.

^*^ *p* Value <.05.

^†^ *p* Value <.01.

To understand the association between loneliness, well-being, and e-Health literacy [RQ1] a Pearson’s correlation test was performed to evaluate the relationship between eHealth literacy, loneliness, and well-being across HI and MI countries (Table 3). Across HI countries, a strong negative correlation between loneliness and well-being scores (*r*(1422) = −0.59, *p* value < .01) was observed. Additional observations identified a very weak negative correlation between loneliness and e-Health literacy scores (*r*(1422) = −0.13, *p* value < .01), and a weak positive correlation between well-being and e-Health literacy scores (*r*(1422) = −0.21, *p* value < .01). Across MI countries, a strong negative correlation between loneliness and well-being scores (*r*(477) = −0.59, *p* value < .01), and a weak positive correlation between well-being and eHealth literacy scores (*r*(477) = −0.15, *p* value < .01). While statistically insignificant, a very weak negative correlation between loneliness and eHealth literacy scores (*r*(477) = −0.07, *p* value = .12) was observed.

To understand the association of well-being across MI and HI countries, [RQ2] respondents (18–29 years) reported the lowest well-being scores (HI = 89.5 ± 13.6, MI = 87.5 ± 13.8) and varied significantly (*p* value < .05) from older adults (40–49, 50–59, and 60+ years). Education levels had a significant impact on well-being in HI (*p* value < .01) and MI (*p* value = .02) countries. Across both countries’ income levels, respondents with a graduate degree (Master’s, PhD) reported the highest well-being scores varying significantly (*p* value < .05) from respondents with less than a high school degree, a college diploma/some college or university. Gender had a significant impact on well-being in HI (*p* value = .03) and MI (*p* value < .01) countries; female respondents reporting higher scores than the male respondents (HI = 94.9 ± 13.9, MI = 93.6 ± 12.8). Marital status had a significant impact on well-being scores across both HI (*p* value < .01) and MI countries (*p* value < .01). Respondents who reported being married/having a partner had higher scores than the single respondents (HI = 95.4 ± 13.7, MI = 95.1 ± 12.1). In HI countries, the community of residence significantly affected well-being, whereby respondents from small towns reported the lowest scores (91.6 ± 15.5).

Additional analysis was conducted to understand the relationship of loneliness, and findings showed marital status was associated with loneliness scores in HI (*p* value < .01) and MI (*p* value = .02) countries. Respondents who reported being married/having a partner (HI = 45.3 ± 5.0, MI = 46.4 ± 4.7) had lower loneliness scores than single respondents. Employment status had a significant impact on loneliness in HI (*p* value = .02) and MI (*p* value = .02) countries, whereby unemployed respondents (HI = 47.8 ± 5.3, MI = 48.0 ± 4.7) reported significantly higher loneliness. In HI countries, education level significantly affected loneliness (*p* value = .02). Respondents who reported having less than a high school degree and respondents with some college diploma/some college or university reported the highest scores. While statistically insignificant, a similar observation was noted in MI countries, whereby respondents with the same degree levels reported the highest loneliness scores. In MI countries, gender had a significant impact on loneliness (*p* value = .02), males had higher scores than female respondents. In MI countries, age had a significant impact on loneliness (*p* value < .01), whereby respondents (18–29 years) reported having the highest scores and varied from older respondents (40–49, 50–59, and 60+ years).

Investigations regarding eHEALS and RQ2 show there are no sociodemographic predictors that had a significant impact on eHEALS scores. Examining HI countries, all predictors except marital status and the type of community (rural/urban/city) showed significant impacts on eHEALS scores. Respondents’ age (50+ years) in HI countries showed a higher significant (*p* value < .01) impact than those <50 years. Education level had a significant impact (*p* value < .01), with respondents with a graduate degree (Master’s, PhD) reporting the highest scores and varied significantly from respondents with less than a Bachelor’s degree. The lowest scores were reported among those with a high school degree or lower. Gender had a significant impact (*p* value = .03), with female respondents reporting higher scores than male respondents. Employment status had a significant impact (*p* value = .04); those employed had the highest eHEALS scores.

The effect of time on loneliness, well-being and e-heals score was investigated because the survey was deployed across two different time periods. Analysis shows 13.9% of the total data were collected during Wave 2, and 84.1% in Wave 1. Data pertaining to HI were primarily collected in Wave 2. The primary comparison in this research is HI versus MI and Wave 2 did not include MI countries, adding time as a variable in the current regression model was impracticable. However, to validate our findings, we performed an analysis after removing the data from Wave 2 and observed similar findings (similarities and differences) between HI and MI countries.

## Discussion

The COVID-19 pandemic had a notable effect on the psychological well-being of individuals across both HI and MI countries. Findings in our study further support that loneliness was an increasingly common experience during the COVID-19 pandemic across nations (Akhter-Khan et al., 2023; Nguyen et al., 2020). While there were many similarities, the experiences of citizens differed across groups. Notably, education, gender, and marital status emerged as strongly associated with loneliness, however, these were not experienced the same across HI and MI countries. This supports the notion that loneliness is influenced by culture and reinforces the value and importance of multinational studies, which can enhance understanding of these differences at a global level. Our findings support the current literature on social loneliness (see, e.g., Barreto et al., 2021) showing that people from different cultures might experience it in different ways. While Barreto and colleagues have indicated the fact that individualist-collectivist values are important in explaining such cultural differences, we draw attention to the country’s economic development.

eHealth literacy during the COVID-19 pandemic was strongly associated with education. Individuals with graduate-level education (e.g., PhD and Master’s) were more likely to report greater eHealth literacy and greater well-being than people with undergraduate and diploma-level training (e.g., college or university). However, this association was stronger in MI countries. A strong association was noted among those who were employed full-time. This may be due to a common requirement for remote communication with others for work-related reasons during the pandemic. Remote communication is a protective factor for loneliness when communicating with friends, yet it was not associated with communication with family members (Arakawa et al., 2023). As friendships in the workplace are common, this may be one reason why employment showed a strong association with loneliness.

Furthermore, the e-heals scores were higher in HI countries than in MI countries showing the fact that country development could play a role in the way people converted online information into positive outcomes for their health during COVID-19 pandemic. Here it is important to acknowledge the fact that our sample consists of participants who already had a relatively good level of digital literacy, as the survey has been conducted online in all countries. The fact that we investigated only the population who was online and could answer an online survey is reflected in the structure of the sample—with people below 40 years of age being overrepresented. Therefore, one of the key limitations is generalizing our findings relating to participants with very low digital literacy.

Marital status was associated with loneliness in both HI and MI countries. Similar to findings in the literature, respondents who reported being married/having a partner in our study reported lower levels of loneliness compared to single respondents (Liu et al. 2021), suggesting that “marital advantage” reduced the risk of loneliness during the pandemic as previously noted by Liu et al. (2023). However, Delaruelle et al. (2023) note it is important not to classify all individuals living alone as all the same, especially for the older adult population, as marital history and diversity in life experiences have been shown to affect loneliness differently.

It has been noted that technology was a panacea for mitigating social isolation and loneliness during the COVID-19 pandemic and times of social distancing policies (Gabbiadini et al., 2020; Robbins et al., 2023). Negative implications associated with digital exclusion including increased risk of loneliness (Nguyen et al., 2020) are further amplified by the mistaken assumption that all individuals in HI and MI countries can access high-quality connectivity and digital devices if they choose to do so. This may be reflected in the age distribution as well as the high levels of education among respondents in the current study. This misconception is often associated with older adults and the perspective that nonusers older than the age of 65 must be technophobic, which leads to stigma, and further exacerbates loneliness (Freeman et al., 2022).

The findings in this study highlight the complexity of examining the role that technology had in influencing and enabling opportunities for engagement and participation in social life, a recognized dimension of successful aging. The intersectoral experiences of individuals during the COVID-19 pandemic cannot be understated as social factors including e-Health literacy and well-being were not uniformly experienced across environments including rural and urban areas as well as across high- and middle-income countries. This greatly supports the revised model of successful aging as posited by Rowe and Khan (2015) as the growing integration of technology into the daily lives of citizens can be a strong influence. Further, the use of technology for virtual communication with others emerged as protective against loneliness. It is therefore important to recognize its value in mitigating environmental contexts to connect individuals across geographic distances, an experience magnified during the pandemic. While experienced differently, findings from this research emphasize the role of technology in enhancing cognitive and social abilities (Shah et al., 2021).

## Strengths and Limitations

The strength of this work lies in the magnitude of the data set, leading to findings about loneliness, well-being, and eHealth across HI and MI countries. The emerging findings contribute original insights into different age cohorts, education levels, marital status, types of community, and gender. The findings presented here are novel, intersecting across different societal factors and academic disciplines, including gerontology, social policy, and gerontechnology, and have implications for current discourse surrounding loneliness, well-being, eHealth, and digital literacy interventions. There are several limitations of this work, including the need for more data collection pertaining to ethnicity and from low-income countries. Our findings indicate that factors such as gender, age, higher education, and income level are associated with greater well-being. The role of such factors needs further exploration in studies regarding e-Health, loneliness, and well-being, beyond the COVID-19 pandemic context.

It is important to note that our sample was nonprobabilistic, with approximately 70% of participants being female, only 13.5% aged 60 or above, 90% reporting having a degree or more advanced studies, and only around 15% were from MI countries. Such differences in sample sizes across MI and HI countries may have affected results. Further studies would require representative samples.

The sample composition, largely representing Europe and North America, may have biased our results, which might have been different if data had been collected from low-income countries. If our sample had included a more balanced representation, with fewer females, more older adults, lower education levels, and a greater proportion of participants from MI and LI countries, our findings might have differed. In addition, further studies should include low-income countries, an important addition to understand the role of country development in the rhetoric of successful aging.

Our recruitment method via social media may have introduced further bias, favoring individuals with Internet access and potentially higher socioeconomic status. The limitations of our study lie in the characteristics of our sample and its potential lack of representativeness. Based on the findings from the systematic review by Oh and colleagues (2021), who highlighted several types of e-Health instruments, the respective authors note the eHEALS tool has received criticism (Collins et al., 2012; Hu et al., 2012; Jordan et al., 2011); specifically, the “lack of an explicit definition of the concept that many health literacy indices were developed to measure limit […] its ability to make fully informed judgments […] about a person’s ability to seek, understand, and use health information” (p. 10). However, a strength from these findings presented here has been identified because we have contributed findings based on data collected from a wider breadth of countries during the pandemic at specific time points. Therefore, the results presented here demonstrate how the eHEALS tool is sensitive to identifying the intricacies of eHealth literacy amongst older people living in other countries not previously identified in existing literature.

Another potential limitation is that the attrition rate of 40% is high, prior studies have reported that, according to the American Psychological Association, American Association of Colleges of Pharmacy, and other organizations, 60% is considered acceptable, especially while conducting multisite studies (Fincham, 2008; National Social Norms Center, 2014). Additionally, the survey response of 60% is well above the average online survey response rate of 44.1% (Wu et al., 2022). Moreover, this survey research had more than 65 questions, making it a relatively longer survey. Moreover, while not common in survey research, a post hoc (retrospective) power analysis revealed that ~500 participants would be sufficient for estimating the differences with seven predictor variables to achieve a power of 0.95. Finally, for the analysis, we did not use a listwise deletion approach because the sociodemographic questions (age, gender, etc.) were the last among the list of questions in the survey, and those excluded (40%) did not have any sociodemographic questions answered making it impossible to use listwise deletion approach for analysis.

Despite these limitations, our sample is sizable and includes multiple HI and MI countries, thus allowing for robust findings. However, it is essential to interpret these results cautiously, considering the potential biases introduced by the sample composition. Not all individuals have equal access to technology, one current limitation of the current study. As an online survey study design, all participants required a basic level of technology literacy and access to participate in our study.

This consortium was organically formed, responded rapidly to the pandemic, and relied on existing networks; the opportunity to deploy the online survey across LI countries was not possible at that time. This work is a primary, essential point of reference and influential for respective fields, global organizations, and nationwide policymakers to understand successful aging across the life course.

We propose recommendations for scholars, policymakers, and organizations, such as the WHO, aligned to their eHealth directive policies:

1. Plan and deliver modular training for people with lower education levels to improve digital literacy skills in line with people with higher education levels. This approach should be met through intersectoral engagement, local and national government authorities, education institutes, and community initiatives.
2. Digital skill training should be part of upskilling opportunities for unemployed people to increase their eHealth and digital literacy skills, knowledge, and experience.
3. Agencies such as the WHO, which leverage substantial influence on global and regional discourse, should emphasize the importance of education, together with employment and digital skills literacy, within their eHealth directives. This can aid member states in recognizing the importance of relationships and intersectionality to benefit adults across the life course and improve successful aging and well-being.

Internationally, these findings contribute to four of the UN sustainable development goals (SDGs; United Nations, 2023): (1) good health and well-being, (2) quality education, (3) gender equality, and (4) reduced inequalities. Aligning with the WHO programme, The Decade of Healthy Aging 2020–2030 (Rudnicka et al., 2020), these findings can aid member states in implementing appropriate actions to benefit their respective societies. This paper must act as a key resource for academics and policymakers in future pandemic preparedness strategies and contemporary directives and policies.

## Conclusions

Current work brings attention to the vulnerabilities and risks of old age during the COVID-19 pandemic by examining loneliness and well-being in different age groups. By comparing HI and MI countries, we aimed to fill an existing gap in literature on successful aging, where financial resources are considered at the individual (Marston et al., 2024) level, but not as structural factors (i.e., country development). Consistent with the existing literature on successful aging and well-being later in life, current research shows that successful agers are younger, more educated, and affluent individuals from HI countries. Still, regardless of the country’s income, lower well-being, and higher loneliness were experienced more by older individuals with poorer socioeconomic resources and lower social capital, showing that the successful aging concept reinforces the discussion of how social inequalities shape the way we age, including people’s resilience in critical times.

## Supplementary Material

gnae170_suppl_Supplementary_Material

## Data Availability

Data used in this study are not available at present because planned analyses for future publications have not been completed. This study was not preregistered.
