# Supplementary material for: Successful Aging Across Middle Versus High-Income Countries: An Analysis of the Role of eHealth Literacy Associated With Loneliness and Well-Being
Source: Gerontologist. 2024 Dec 14;65(1):gnae170. doi: 10.1093/geront/gnae170 (PMC11712271; doi:10.1093/geront/gnae170)
Supplement: gnae170_suppl_Supplementary_Material [file gnae170_suppl_supplementary_material.docx]

**Supplementary Material**

Supplementary Figure 1. Overview of Measurements.

- Computer use and behaviour
- Purpose of using technology (e.g., using social media to communicate, and challenges faced)

Section A

- COVID-19 related questions

Section B

- Activities of daily living during COVID-19.
- Social connections and friendships
- Key worker responsibilities

Section C

- Psychological wellbeing
- Autonomy and Personal Growth
- Environmental Mastery
- Positive Relations with Others
- Purpose in Life and Self-Acceptance

Section D

- eHealth and digital literacy

Section E

- Loneliness
- UCLA Loneliness Scale version 3.

Section F

- Digital software technologies (e.g., mobile app, SMS etc.]

Section G

- Demographics
- Age, gender, education, marital status
- Number of people staying in the same household
- Employment status
- Geographic location

Section H
